# Targeting Photoreceptors via Intravitreal Delivery Using Novel, Capsid-Mutated AAV Vectors

**DOI:** 10.1371/journal.pone.0062097

**Published:** 2013-04-26

**Authors:** Christine N. Kay, Renee C. Ryals, George V. Aslanidi, Seok Hong Min, Qing Ruan, Jingfen Sun, Frank M. Dyka, Daniel Kasuga, Andrea E. Ayala, Kim Van Vliet, Mavis Agbandje-McKenna, William W. Hauswirth, Sanford L. Boye, Shannon E. Boye

**Affiliations:** 1 Department of Ophthalmology, University of Florida College of Medicine, Gainesville, Florida, United States of America; 2 Division of Cellular and Molecular Therapy, Department of Pediatrics, University of Florida College of Medicine, Gainesville, Florida, United States of America; 3 Department of Biochemistry and Molecular Biology, University of Florida, College of Medicine, Gainesville, Florida, United States of America; 4 Department of Molecular Genetics and Microbiology, University of Florida College of Medicine, Gainesville, Florida, United States of America; University of Kansas Medical Center, United States of America

## Abstract

Development of viral vectors capable of transducing photoreceptors by less invasive methods than subretinal injection would provide a major advancement in retinal gene therapy. We sought to develop novel AAV vectors optimized for photoreceptor transduction following intravitreal delivery and to develop methodology for quantifying this transduction *in vivo*. Surface exposed tyrosine (Y) and threonine (T) residues on the capsids of AAV2, AAV5 and AAV8 were changed to phenylalanine (F) and valine (V), respectively. Transduction efficiencies of self-complimentary, capsid-mutant and unmodified AAV vectors containing the smCBA promoter and mCherry cDNA were initially scored *in vitro* using a cone photoreceptor cell line. Capsid mutants exhibiting the highest transduction efficiencies relative to unmodified vectors were then injected intravitreally into transgenic mice constitutively expressing a Rhodopsin-GFP fusion protein in rod photoreceptors (Rho-GFP mice). Photoreceptor transduction was quantified by fluorescent activated cell sorting (FACS) by counting cells positive for both GFP and mCherry. To explore the utility of the capsid mutants, standard, (non-self-complementary) AAV vectors containing the human rhodopsin kinase promoter (hGRK1) were made. Vectors were intravitreally injected in wildtype mice to assess whether efficient expression exclusive to photoreceptors was achievable. To restrict off-target expression in cells of the inner and middle retina, subsequent vectors incorporated multiple target sequences for miR181, an miRNA endogenously expressed in the inner and middle retina. Results showed that AAV2 containing four Y to F mutations combined with a single T to V mutation (quadY−F+T−V) transduced photoreceptors most efficiently. Robust photoreceptor expression was mediated by AAV2(quadY−F+T−V) −hGRK1−GFP. Observed off-target expression was reduced by incorporating target sequence for a miRNA highly expressed in inner/middle retina, miR181c. Thus we have identified a novel AAV vector capable of transducing photoreceptors following intravitreal delivery to mouse. Furthermore, we describe a robust methodology for quantifying photoreceptor transduction from intravitreally delivered AAV vectors.

## Introduction

Clinical trials for RPE65-Leber congenital amaurosis (LCA) have demonstrated the ability to deliver therapeutic transgene to the retinal pigment epithelium (RPE) by subretinal injection thereby restoring retinal function and visually-evoked behavior to patients [1]–[3]. Given the predominance of photoreceptor (PR) specific retinal degenerations [4], there is a need to develop PR targeted gene therapies. Of equal importance is the need to develop a less invasive vector delivery procedure than subretinal injection, particularly when an underlying genetic defect results in an atrophic retina vulnerable to further damage following surgically induced retinal detachment. Jacobson et al. reported that subretinal injection under a cone-rich fovea resulted in decreased visual acuity (in some LCA2 patients) and foveal thinning (in both LCA2 patients and non-human primates) [5], [6]. Development of viral vectors capable of transducing PRs via an intravitreal approach would provide an ideal therapeutic option for retinal degeneration patients.

Adeno-associated virus (AAV) is considered the optimal vector for ocular gene therapy due to its efficiency, persistence and low immunogenicity [7]. Identifying vectors capable of transducing PRs via the vitreous will rely partially on identifying which serotypes have native tropism for this cell type following local delivery. Several serotypes have been used to successfully target transgene to PRs following subretinal injection, including AAV2, AAV5 and AAV8 with all three demonstrating efficacy in proof of concept experiments across multiple species (mouse, rat, dog, pig and non-human primate) [8]–[21]. Studies comparing their relative efficiency following subretinal delivery in the rodent show that both AAV5 and AAV8 transduce PRs more efficiently than AAV2, with AAV8 being the most efficient [11], [15], [22]–[24]. We previously showed that AAV2 and AAV8 vectors containing point mutations of surface-exposed tyrosine residues (tyrosine to phenylalanine,Y−F) display increased transgene expression in a variety of retinal cell types relative to unmodified vectors following both subretinal and intravitreal injection [25], [26]. Of the vectors tested, AAV2 triple mutant (triple Y−F) exhibited the highest transduction efficiency following intravitreal injection whereas the quadruple mutant (quad Y−F) exhibited the novel property of enhanced transduction of outer retina [26]. Further improvements in transduction efficiency may be achieved via directed mutagenesis of surface exposed threonine (T) residues to either valine (V) or alanine (A). Both Y−F and T−V/T−A mutations increase efficiency by decreasing phosphorylation of capsid and subsequent ubiquitination as part of the proteosomal degradation pathway [27]–[29]. In this study, we compare AAV2, AAV5 and AAV8-based vectors containing a combination of Y and T mutations for their ability to transduce PRs following intravitreal injection.

We have found that the transduction profile of intravitreally-delivered AAV is heavily dependent upon the injection procedure itself. Due to the small size of the mouse eye, it is not uncommon for trans-scleral, intravitreal injections to result in damage to the retina that might allow delivery of some vector directly to the subretinal space. In this study, we describe our method for reducing this damage, thus preventing injection variability and allowing for accurate comparisons to be made among vectors. Furthermore, we describe a novel method for quantifying transduction efficiency *in vivo* using knock-in mice bearing a human rhodopsin-enhanced green fluorescent protein (EGFP) fusion gene (RhoGFP mice) [30], AAV vectors driving mCherry, and subsequent fluorescent activated cell sorting (FACS) to quantify both ‘on-target’ PR transduction (GFP and mCherry positive cell population) and ‘off-target’ retinal cell types (GFP negative, mCherry positive cell population). This method for scoring intravitreally-delivered, AAV- mediated PR transduction can be applied toward development of additional vectors intended for the treatment of inherited retinal disease.

With the enhanced serotypes we identified, we sought to reduce off-target transgene expression by incorporating the human rhodopsin kinase (hGRK1) promoter in vectors. hGRK1 has demonstrated PR exclusive transduction when incorporated into AAV vectors delivered subretinally to mice and non-human primates [18], [31]. Similar to methods previously described, [32] we further restricted transgene expression to PRs by incorporating multiple target sequences for miR181, an miRNA endogenously expressed in cells of the inner and middle retina.

## Methods

### Vector production

The following vector plasmid constructs were cloned and packaged in unmodified AAV serotypes 2, 5, and 8 and capsid mutant derivatives of these serotypes; self-complementary small chicken ß-actin driving mCherry (sc-smCBA-mCherry), standard (non self-complimentary) human rhodopsin kinase driving green fluorescent protein (hGRK1−GFP), and standard full length chicken ß-actin driving GFP (CBA-GFP). Promoter constructs were identical to those previously described [31], [33], [34]. A hGRK1-GFP-miR181c construct was also generated and packaged in AAV2(quad Y−F+T−V) by inserting four tandem copies of complementary sequence for mature miR-181 (5′ ACTCACCGACAGGTTGAA 3′) (Atlas of miRNA distribution: http://mirneye.tigem.it/) immediately downstream of GFP, similar to Karali et al. [32].

AAV2, AAV5 and AAV8 capsid mutants were generated by directed mutagenesis of surface-exposed tyrosine and threonine residues with the QuickChange Multi Site-Directed Mutagenesis Kit (Agilent Technologies, CA 200514). Selected tyrosine residues were mutated to phenylalanine (Y−F) whereas threonine residues were mutated to valine (T−V) [27]. Table 1 describes amino acid location of mutations for experimental mutant vectors. All vectors were packaged, purified, and titered according to previously described methods [6], [35].

**Table 1:** Nomenclature for capsid-mutated vectors with description of amino acid location of mutation.

Vector nomenclature	Mutation
AAV2(tripleY-F)	Y275F+Y447F+Y733F
AAV2(tripleY-F+T-V)	Y275F+Y447F+Y733F +T491V
AAV2(quadY-F)	Y272F+Y444F+Y500F+Y730F
AAV2(quadY-F+T-V)	Y272F+Y444F+Y500F+Y730F+T491V
AAV5(singleY-F)	Y719F
AAV5(doubleY-F)	Y263F+Y719F
AAV8(doubleY-F)	Y447F+Y733F
AAV8(doubleY-F+T-V)	Y447F+Y733F+T494V

### Cell lines

661W cone cells[36] (generously provided by Dr. Muayyad R. Al-Ubaidi, University of Oklahoma Health Sciences Center, Oklahoma City, OK) were passaged by dissociation in 0.05% (w/v) trypsin and 0.02% (w/v) EDTA, followed by replating at a split ratio ranging from 1:3 to 1:5 in T75 flasks [37]. Cells were maintained in DMEM containing 10% FBS, 300 mg/l glutamine, 23 mg/l putrescine, 40 µl of ß-mercaptoethanol, and 40 µg of hydrocortisone 21-hemisuccinate and progesterone. The media also contained penicillin (90 units/ml) and streptomycin (0.09 mg/ml). Cultures were incubated at 37°C [38].

### Infections and FACS analysis

661W cells were plated in 96 well plates at a concentration of 1.0×10^4^ cells/well. The following day, cells were infected at 10,000 p/cell with sc-smCBA-mCherry packaged in unmodified and modified AAV2, AAV5 or AAV8 vectors. Three days post-infection, fluorescent microscopy at a fixed exposure was performed, cells were detached and FACS analysis was used to quantify reporter protein (mCherry) fluorescence. Transduction efficiency (mCherry expression) of each AAV vector was calculated as previously reported [37] by multiplying the percentage of positive cells by the mean fluorescence intensity in each sample [23].

### Animals

Vectors were injected in 1 month old C57BL/6 mice (Jackson Laboratory, Bar Harbor, ME) and in 1 month old heterozygote Rho-GFP mice, knock-in mice bearing human rhodopsin-GFP fusion gene (generously provided by Dr. Alecia Gross, University of Alabama at Birmingham).

### Ethics statement

All mice were maintained in the University of Florida Health Science Center's animal care facilities and were handled in accordance with the ARVO statement for Use of Animals in Ophthalmic and Vision Research and the guidelines of the Institutional Animal Care and Use Committee of the University of Florida. Animal work performed in this study was approved by UF's IACUC (animal protocol #201207573).

### Intravitreal injections

Prior to vector administration, mice were anesthetized with ketamine (72 mg/kg)/xylazine (4 mg/kg) by intraperitoneal injection. Eyes were dilated with 1% atropine and 2.5% phenylephrine. 1.5 μl of unmodified or capsid mutated vector was delivered to the intravitreal cavity of adult mice. An aperture was made 0.5 mm posterior to the limbus with a 32-gauge ½ inch needle on a tuberculin syringe (BD, Franklin Lakes, NJ) followed by introduction of a blunt 33-gauge needle on a Hamilton syringe. GenTeal gel, 0.3% (Novartis) was applied to the corneal surface and a glass coverslip was laid onto this interface for visualization through the microscope to guide the needle into the vitreous cavity without retinal or lenticular perforation. Extreme care was taken with this visualization technique to confirm that no retinal perforation occurred.

For experiments evaluating activity of the hGRK1 promoter in C57BL/6 mice, 7.5×10^9^ vg of AAV2-based vectors, 8.5×10^10^ vg of AAV5(singleY−F), 5.3×10^9^ vg of AAV5(doubleY−F), 1.3×10^11^ vg of AAV8(doubleY−F) and 6.0×10^10^ vg of AAV8(doubleY−F+T−V) were delivered. For experiments evaluating the CBA promoter in C57BL/6 mice, all vectors were delivered at a concentration of 1.5×10^10^ vg. To evaluate transduction of vectors containing microRNA target sequence in C57BL/6 mice, a concentration of 1.5×10^10^ vg was used. All Rho-GFP mice were injected intravitreally with 1.5×10^9^ vg.

### Fundoscopy

At 4 weeks post-injection, fundoscopy was performed using a using a Micron III camera (Phoenix Research Laboratories, Pleasanton, CA). Bright field, green fluorescent and red fluorescent images were taken to visualize retinal health, GFP expression and mCherry expression, respectively. Exposure settings were constant between experiments.

### Retinal dissociation and FACS analysis

4 weeks post injection, Rho-GFP retinas were harvested and dissociated with the papain dissociation system (Worthington Biochemical Corporation, NJ, Cat #3150). FACS analysis was used to quantify the percentage of cells that were GFP positive (PRs), mCherry positive (any retinal cells transduced with vector) and both GFP and mCherry positive (PRs transduced by vector). The percentage of mCherry positive PRs was calculated as the ratio of cells both GFP and mCherry positive relative to total GFP positive PRs.

### Immunohistochemistry (IHC)

Immediately after fundoscopy, eyes were enucleated and tissue was prepared for cryoprotection and sectioning as previously described [23]. Briefly, after rinsing with 1X PBS, sections were incubated with 0.5% Triton X−100 for 1 hour followed by a 30 minute incubation with a blocking solution of 1% bovine serum albumin (BSA). Retinal sections were then incubated overnight at 4°C in a rabbit polyclonal antibody raised against GFP (generously provided by Dr. Clay Smith; University of Florida, Gainesville, Florida) diluted in 0.3% Triton X−100/1% BSA at 1:1,000. The following day, sections were rinsed with 1X PBS and incubated for one hour at room temperature in anti-rabbit IgG secondary antibody Alexa-fluor 488 (Invitrogen, Eugene, Oregon, Cat#A11008) diluted in 1X PBS at 1:500. Finally, sections were counterstained with 4′,6′-diaminio-2-phenylindole (DAPI) for 5 minutes at room temperature. Retinal sections were imaged using a fluorescent Axiophot microscope (Zeiss, Thornwood, NY) as previously described [23]. Images were captured at 5X, 20X and 40X. Exposure settings were consistent across images at each magnification.

A semi-quantitative comparison of the number of GFP-positive photoreceptors was made between eyes injected intravitreally with either AAV2(quadY−F+T−V)−hGRK1−GFP or AAV2(quadY−F+T−V)−CBA−GFP (identical titers) by counting GFP-positive photoreceptors in representative sections. Low magnification (merged, 10X) and high magnification (40X) images were taken. Cell counts were made in 4 anatomically matched areas of each representative retina. Each respective area was uniform in size by virtue of magnification (40X) and contained on average 30 columns of photoreceptor cell bodies. Results were plotted in Sigma Plot for graphical presentation.

## Results

### Quantification of in vitro transduction efficiency

**Figure 1:**
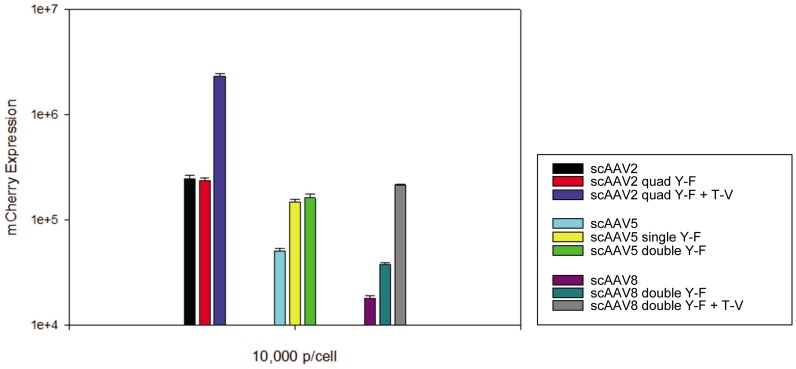
Transduction efficiency of unmodified and capsid mutated vectors *in vitro* **.** 661W cells were infected with scAAV2, scAAV2(quadY-F), scAAV2(quadY-F +T-V), scAAV5, scAAV5(singleY-F), scAAV5(doubleY-F), and scAAV8, scAAV8(doubleY-F), and scAAV8(doubleY-F+T-V) at a multiplicity of infection (MOI) of 10,000. mCherry expression is shown in arbitrary units on the ‘y’ axis, calculated by multiplying the percentage of positive cells by the mean fluorescence intensity in each sample.

661W mouse cone PR cells were infected with unmodified or capsid mutated, self-complimentary AAV vectors containing the smCBA promoter driving mCherry in order to quantify relative transduction efficiencies of all vectors. FACS analysis provided a measure of relative transduction efficiency (mCherry expression) across samples. Figure 1 shows mCherry expression, in arbitrary units, for each capsid tested; scAAV2, scAAV2(quadY−F), scAAV2(quadY−F+T−V), scAAV5, scAAV5(singleY−F), scAAV5(doubleY−F), scAAV8, scAAV8(doubleY−F), scAAV8(doubleY−F+T−V). This preliminary screen revealed that AAV2(quadY−F+T−V) transduced cone cells most efficiently. Increases in AAV2(quadY−F+T−V) mediated mCherry expression were ∼10 fold above the scAAV2 baseline (Figure 1). scAAV8 transduced 661W cells least efficiently.

### Quantification of in vivo transduction efficiency

**Figure 2:**
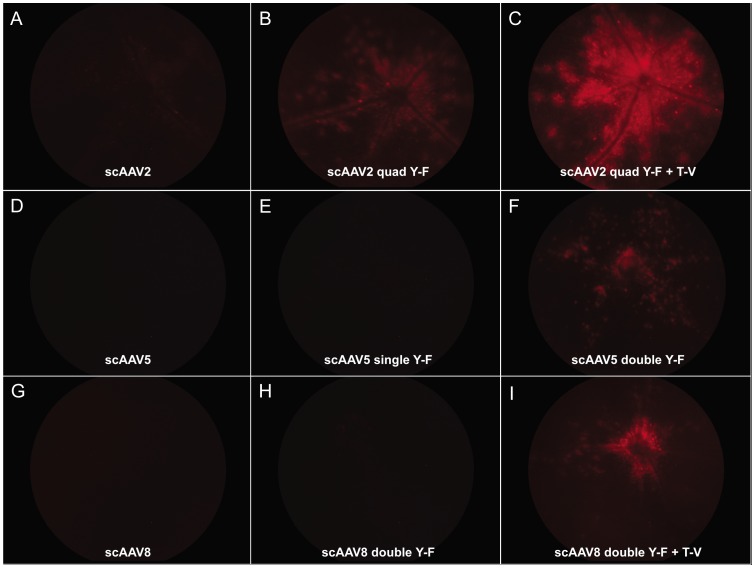
Qualitative comparison of unmodified and capsid mutated AAV vectors *in vivo* **.** Fundoscopy (red channel only) of Rho-GFP mice 4 weeks post-injection with unmodified and capsid-mutated scAAV-smCBA-mCherry vectors (1.5×10^9^ vg delivered). Exposure and gain settings were the same across all images.

**Figure 3:**
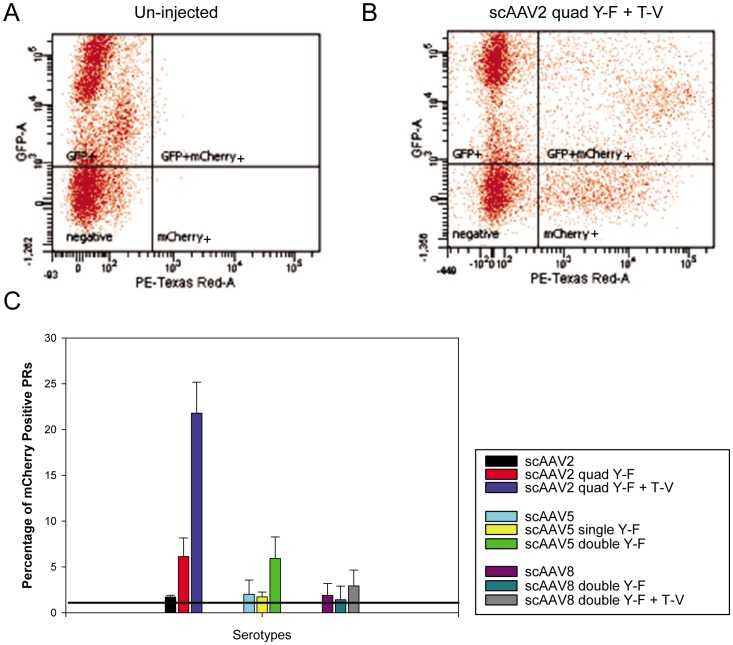
Quantitative comparison of unmodified and capsid mutated AAV vectors *in vivo* **.** Transduction efficiency of unmodified and capsid-mutated scAAV2, scAAV5 and scAAV8 vectors in Rho-GFP mice. FACS analysis was used to quantify the percentage of cells that were GFP positive (PRs), mCherry positive (any retinal cells transduced with AAV) and both GFP and mCherry positive (PRs transduced by AAV). Representative plots for a negative control (uninjected retina) and 2 pooled retinas injected with scAAV2(quadY-F+T-V) are shown in Panels A and B, respectively. Cells that were both GFP and mCherry positive are shown in the top right of Panels A and B and represent the percent of transduced PRs. The bottom right of Panels A and B show cells that were mCherry positive but GFP negative, representing off-target transduction. The percentage of mCherry positive PRs (a measure of *in vivo* PR transduction efficiency for each vector) in retinas injected with unmodified or capsid-mutated scAAV vectors is shown in Panel C.

Following *in vitro* screening, identical vectors were evaluated for their relative ability to transduce PRs *in vivo* following intravitreal injection in 1 month old, heterozygote Rho-GFP mice (1.5×10^9^ vg delivered). Fundoscopy at 4 weeks post-injection showed qualitatively that mCherry expression was enhanced with addition of capsid mutations to each serotype (Figure 2). Rho-GFP mouse retinas injected intravitreally with scAAV2(quadY−F+T−V) −smCBA−mCherry exhibited the highest qualitative levels of mCherry expression (Figure 2C). Levels of transgene expression achieved following intravitreal injection of scAAV2(quadY−F), scAAV5(doubleY−F) and scAAV8(doubleY−F+T−V) were approximately equivalent. To quantify the relative ability of each vector to transduce PRs, intravitreally injected Rho-GFP retinas were dissociated and FACS analysis performed. Cells were sorted into four populations: 1) non-fluorescent: indicating un-transduced, non-PR retinal cells (“negative”), 2) green fluorescent only: indicating untransduced PRs (“GFP+”), 3) red and green fluorescent: indicating transduced PRs (“GFP+mCherry+”) and 4) red fluorescent only: indicating transduced non-PR retinal cells (“mCherry+”) (Figure 3). As shown in Figure 3A and B, an un-injected Rho-GFP retina contains two populations of cells (“GFP+” representing PRs and “negative” representing non-PRs) whereas a Rho-GFP retina injected with scAAV2(quadY−F+T−V) contains all four populations of cells. The relative percentage of mCherry-positive PRs following intravitreal injection of all vectors is shown in Figure 3C. Addition of quadY−F and quadY−F+T−V mutations to the AAV2 capsid surface resulted in ∼3.5 fold and ∼13 fold increases in the percentage of mCherry positive PRs, respectively. Unmodified scAAV2 transduced 1.7% of PRs from the vitreous whereas scAAV2(quadY−F) and scAAV2(quadY−F+T−V) transduced 6.1% and 21.8%, respectively. scAAV2(quadY−F+T−V) transduced the highest number of PRs of all vectors tested. Retinas injected with unmodified and modified AAV5 and AAV8-based vectors exhibited lower efficiencies of PR transduction. Consistent with fundoscopic observations, appreciable PR transduction was seen following intravitreal injection of scAAV2(quadY−F), scAAV5(doubleY−F) and scAAV8(doubleY−F+T−V). The percent of mCherry positive PRs in retinas injected with scAAV5, scAAV5(singleY−F) and scAAV5(doubleY−F) was 2.0%, 1.7% and 5.9%, respectively. The percent of mCherry positive PRs in retinas injected with scAAV8, scAAV8(doubleY−F) and scAAV8(doubleY−F+T−V) was 1.9%, 1.4% and 2.9%, respectively. We also found that quantitative comparisons could be made using this methodology at just 1 week post intravitreal injection with scAAV2-based vectors (Figure S1). While fewer total PRs expressed detectable levels of mCherry at this early time point, the pattern remained the same, with scAAV2(quadY−F+T−V) mediating the highest levels of transgene expression in PRs.

### Qualitative analysis of photoreceptor transduction

**Figure 4:**
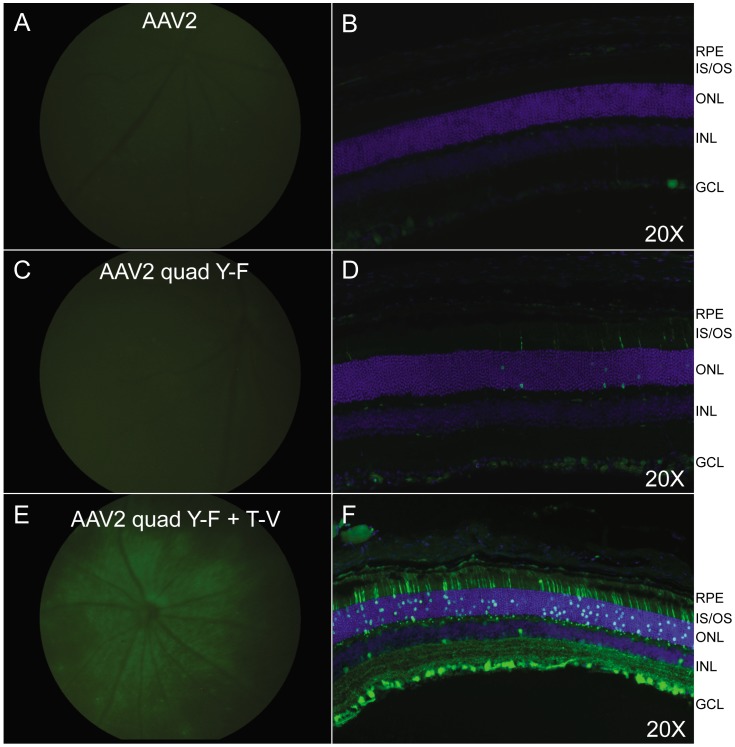
*In vivo* analysis of AAV2-based vectors containing the hGRK1 promoter. Fundus images paired with immunohistochemistry of frozen retinal cross-sections from C57BL/6 mice taken 4 weeks post injection with AAV2, AAV2(quad Y-F), and AAV2(quad Y-F +T-V) vectors containing hGRK1-GFP (7.5×10^9^ vg delivered). Identical gain and exposures were used for fundoscopy. All tissue sections were imaged at 20X, with identical gain and exposure settings. GFP expression is shown in green. Nuclei were counterstained with DAPI (blue). RPE- retinal pigment epithelium, IS/OS- inner segments/outer segments, ONL- outer nuclear layer, INL- inner nuclear layer, GCL- ganglion cell layer.

With the intention to restrict transgene expression to PRs following intravitreal delivery of AAV, we incorporated the PR-specific hGRK1 promoter into unmodified and capsid-mutated vectors. Because our interest lies in evaluating vectors that are relevant for treatment of inherited retinal disease (i.e. those that can accommodate promoter and transgene sequence likely too large to package as self-complementary AAV), all vectors in this set of experiments were single stranded, i.e. non self-complementary. Representative fundus images of C57BL/6 mice and their immunostained retinal sections taken 4 weeks post-intravitreal injection with AAV2, AAV2(quadY−F) and AAV2(quadY−F+T−V) are shown in Figure 4 (7.5×10^9^ vg delivered for all vectors.) Consistent with the quantification results shown in Figure 3, very few PRs expressed GFP following intravitreal injection of AAV2 or AAV2(quad Y−F) (Figure 4B,D). However, robust GFP expression was seen in the PRs following injection of AAV2(quadY−F +T−V) (Figure 4F). AAV2(quadY−F+T−V)−mediated transgene expression was evident in PRs throughout the retina rather than in one specific location. This representative section, in conjunction with surgical observations and fundoscopy support the premise that our injection procedure did not involve retinal perforation and was, in fact, intravitreal (Figure S2). Although reports have shown that the hGRK1 promoter has exclusive activity in rods and cones of mouse and non-human primate when incorporated into subretinally-delivered AAV [18], [31] we observed hGRK1-mediated transgene expression in ganglion cells of injected mice (Figure 4D,F).

**Figure 5:**
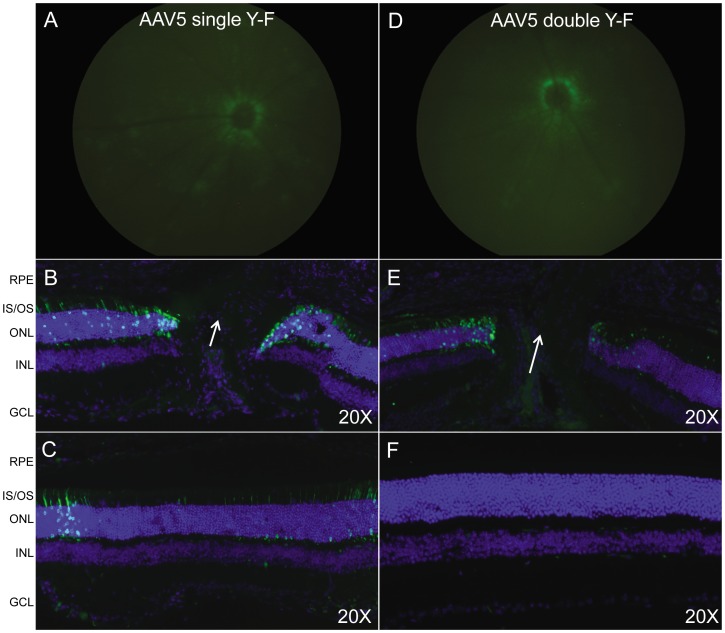
*In vivo* analysis of AAV5-based vectors containing the hGRK1 promoter. Fundus images paired with IHC of frozen retinal cross-sections from C57BL/6 mice taken 4 weeks post injection with capsid mutated AAV5 vectors containing hGRK1-GFP. For analysis of AAV5(singleY-F) and AAV5(doubleY-F) vectors 8.5×10^10^ vg and 5.3×10^9^ vg were delivered, respectively. Retinal tissue sections containing optic nerve head (Panels B and E) and peripheral retinal cross sections (Panels C and F) are shown. White arrows demarcate the optic nerve head. Identical gain and exposures were used for fundoscopy. All cross sections were imaged at 20X, with identical gain and exposure settings. GFP expression is shown in green. Nuclei were counterstained with DAPI (blue). RPE- retinal pigment epithelium, IS/OS- inner segments/outer segments, ONL- outer nuclear layer, INL- inner nuclear layer, GCL- ganglion cell layer.

**Figure 6:**
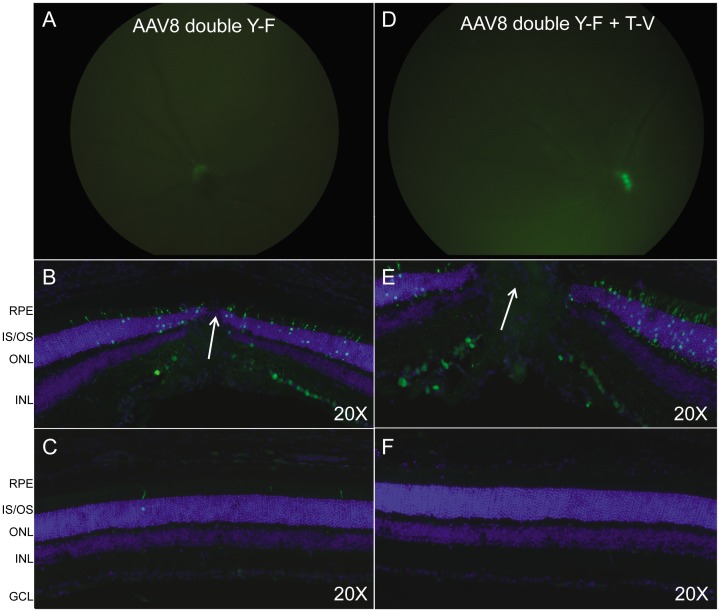
*In vivo* analysis of AAV8-based vectors containing the hGRK1 promoter. Fundus images paired with immunohistochemistry of frozen retinal cross-sections from C57BL/6 mice taken 4 weeks post injection with capsid mutated AAV8 vectors containing hGRK1-GFP. For analysis of AAV8(doubleY-F) and AAV8(doubleY-F+T-V) vectors, 1.3×10^11^ and 6.0×10^10^ vg were delivered, respectively. Retinal cross sections containing optic nerve head (Panels B and E) and peripheral retinal cross sections (Panels C and F) are shown. White arrows demarcate the optic nerve head. Identical gain and exposures were used for fundoscopy. All cross sections were imaged at 20X, with identical gain and exposure settings. GFP expression is shown in green. Nuclei were counterstained with DAPI (blue). RPE- retinal pigment epithelium, IS/OS- inner segments/outer segments, ONL- outer nuclear layer, INL- inner nuclear layer, GCL- ganglion cell layer.

*In vivo* quantification data in Rho-GFP mice revealed relatively low levels of PR transduction following intravitreal delivery of 1.5×10^9^ vg of AAV5- and AAV8-based vectors (Figure 3). Therefore, in order to maximize expression and qualitatively analyze general transduction patterns, higher titers of AAV5- and AAV8-based vectors were used for the following experiments. For analysis of AAV5(singleY−F) and AAV5(doubleY−F) vectors 8.5×10^10^ vg and 5.3×10^9^ vg were delivered, respectively. Fundus images paired with fluorescent images of retinal cross-sections show minimal PR transduction following intravitreal injection of AAV5(singleY−F) and AAV5(doubleY−F) (Fig. 5). A pattern of peripapillary tropism was evident, with PRs around the optic nerve exhibiting the most prominent transgene expression (Figure 5A,B,D,E). PR transduction was found in scattered peripheral retinal sections of AAV5(singleY−F)-injected eyes (Figure 5C), with expression typically found near the retinal vasculature. For analysis of AAV8(doubleY−F) and AAV8(doubleY−F+T−V) 1.3×10^11^ and 6.0×10^10^ vg were delivered, respectively. Fundus images (at 4 weeks post injection) paired with fluorescent images of retinal cross-sections show minimal PR transduction following intravitreal injection of either vector (Figure 6). Similar to AAV5-based vectors, a pattern of peripapillary tropism was seen following injection of modified AAV8 vectors (Figure 6A,B,D,E).

### MicroRNA-mediated regulation of transgene expression

**Figure 7:**
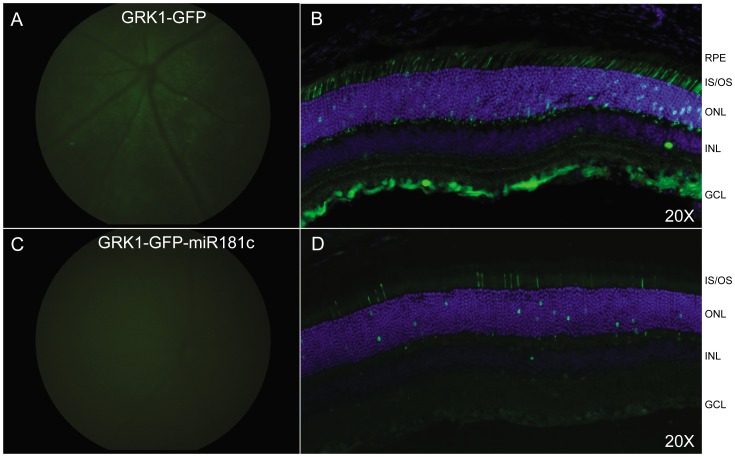
MicroRNA-mediated regulation of transgene expression. Both hGRK1-GFP and hGRK1-GFP-miR181c were packaged in AAV2(quadY-F+T-V) and delivered intravitreally to C57BL/6 mice (1.5×10^10^ vg). Fundoscopy at 4 weeks post injection is shown adjacent to immunohistochemistry of frozen retinal cross-sections. Identical gain and exposures were used for fundoscopy. All cross sections were imaged at 20X, with identical gain and exposure settings. GFP expression is shown in green. Nuclei were counterstained with DAPI (blue). RPE- retinal pigment epithelium, IS/OS- inner segments/outer segments, ONL- outer nuclear layer, INL- inner nuclear layer, GCL- ganglion cell layer.

In order to mitigate the observed off- target transgene expression in ganglion cells following intravitreal delivery of hGRK1-containing AAV vectors, we incorporated a target sequence for miR181, an miRNA shown to be expressed exclusively in ganglion cells and inner retina into our AAV vectors (Atlas of miRNA distribution: http://mirneye.tigem.it/) immediately downstream of GFP, similar to Karali et al.) [32]. The intended effect was to degrade vector derived transcripts and inhibit synthesis of viral-mediated protein in all cells of the retina except PRs. Both hGRK1-GFP and hGRK1-GFP-miR181c were packaged in AAV2(quadY−F+T−V) and delivered intravitreally to C57BL/6 mice (1.5×10^10^ vg). At 4 weeks post-intravitreal injection, funduscopy and IHC on frozen retina cross sections revealed that addition of miR181c to the vector construct did eliminate off-target expression (Figure 7). Although hGRK1-GFP-miR181c-mediated GFP expression was exclusive to PRs, it was also appreciably decreased (Figure 7).

### Qualitative analysis of serotype tropism

Because mutations in genes expressed in retinal cell types other than PRs can also cause or result in retinal degeneration, we incorporated the ubiquitous CBA promoter into vectors to ascertain what other retinal cells types were targeted following intravitreal injection of our strongest capsid-mutated vectors (Figure S4). All vectors were delivered intravitreally at a concentration of 1.5×10^10^ vg. AAV2(quadY−F) and AAV2(quadY−F+T−V) vectors were chosen for testing based on their performance in our PR-targeting experiments described above. AAV2(tripleY−F+T−V) was chosen based on the documented efficiency of AAV2(tripleY−F) in multiple *in vitro* and *in vivo* settings [26], [37]. AAV8(doubleY−F+T−V) was also evaluated. All AAV2-based vectors mediated robust, pan-retinal GFP expression (Figure S4A,B,D,E,G,H), with GFP found throughout the inner and middle retina (Figure S4B,C,E,F,H,I). AAV2(quadY−F+T−V)− and AAV2(tripleY−F+T−V)−mediated GFP expression was also seen in PR cells bodies (Figure S4C,I). AAV8(doubleY−F+T−V)−CBA−GFP exhibited peripapillary tropism ( Figure S3), a pattern similar to that seen with the corresponding hGRK1-containing vector.

A semi-quantitative comparison of photoreceptor transduction following injection of either AAV2(quadY-F+T-V)-CBA-GFP or AAV2(quadY-F+T-V)-hGRK1-GFP was made by counting GFP-positive photoreceptors in 4 representative areas of retina injected with each respective vector. Whole eyecups (merged 10X images) and high magnification (40X) images of representative sections are shown in Figures S5 and S6. GFP positive photoreceptors in retinas injected with AAV2(quadY-F+T-V)-CBA-GFP were distinguished from Muller glia by counting GFP positive cell bodies and outer segments (white arrows, Figure S5A). A comparison of cell counts is presented in Figure S7. GFP-positive photoreceptors were more prevalent throughout the retinas of AAV2(quadY-F+T-V)-hGRK1-GFP-injected mice.

## Discussion

The development of viral vectors capable of efficiently transducing PRs via a less invasive delivery method than the previously utilized subretinal injection route would be a critical advance in retinal gene therapy. In recent years, focus has been placed on identifying novel AAV capsid variants that exhibit increased transduction efficiency and/or altered tropism. To this end, two methodologies have been employed; rational mutagenesis and directed evolution. These approaches have led to identification of novel capsids with increased transduction efficiency [25] altered tropism [26], [39], [40] and the ability to evade recognition by the immune system [41]. “Rational mutagenesis” describes a knowledge-based approach to manipulating the viral capsid to develop customized vectors with distinctive features. Rational mutagenesis of surface-exposed tyrosine, threonine and lysine residues results in increased transduction by decreasing phosphorylation and subsequently reducing ubiquitination and proteosomal degradation of the AAV capsid [27]–[29]. We previously showed that Y−F mutations on the AAV2, AAV8 and AAV9 capsid surface led to increased transduction and altered transduction profiles relative to unmodified vectors following both subretinal and intravitreal delivery [25], [26]. Proof of concept studies later showed that incorporation of these mutations led to more pronounced rescue in animal models of inherited retinal disease [23], [42] and in one case, conferred therapy in a particularly aggressive mouse model that was refractory to treatment using an unmodified parent serotype [24]. Directed evolution can select for desired characteristics without *a priori* knowledge of the physical determinants, allowing identification of novel vectors that exhibit desired, specific tropisms [43]. Directed evolution has been applied to select AAV variants from combinatorial libraries that demonstrate a diverse range of cellular tropisms *in vivo* relative to their parent serotypes [43]. In the retina, this technology was used to identify a variant capable of specifically transducing Muller cells via the vitreous [39].

With the goal to develop vectors capable of transducing PRs via intravitreal delivery, extracellular determinants of viral transduction must also be considered. The internal limiting membrane (ILM) which defines the border between the retina and vitreous acts as a physical and biological barrier to AAV transduction following intravitreal injection in rodent and non-human primate retina [44], [45]. It has been shown that AAV2 and AAV8 attach to the ILM and accumulate at the vitreoretinal junction, with AAV2 exhibiting the most robust attachment [44]. However, only AAV2 mediated detectable transgene expression in the inner retina [44]. AAV2 binds heparan sulfate proteoglycan (HSPG) which is abundant in the ILM [46], [47], while AAV8 binding involves the laminin receptor which may mediate a weaker interaction with this structure [48]. Here we show that addition of Y−F and T−V mutations to the AAV8 capsid modestly improves its ability to transduce inner/middle/outer retina following intravitreal injection demonstrating the importance of both extracellular and intracellular barriers to transduction. Standard AAV5 fails to attach or accumulate at the ILM [44], likely because it relies on sialic acid for initial binding, a monosaccharide absent from the ILM [49], [50]. Removal of this physical barrier with protease, however, led to robust gene expression in various cells of the retina, including PRs and RPE [44]. Similar to AAV8, here we show that addition of Y−F mutations to the AAV5 capsid surface only modestly improves its ability to transduce outer retina following intravitreal delivery. Taken together, it is clear that the cellular receptors of the parent AAV serotype play a key role in influencing vector interaction with this vitreoretinal interface. Our results are consistent with findings that AAV2-based vectors have the highest affinity for the ILM [44] suggesting that, as of now, capsid mutants based on this serotype have the highest potential for targeting transgene to PRs via the vitreous. As the capsid biology of AAV8, a strong transducer of PRs *in situ*, becomes known, an approach that capitalizes on respective receptor biology of AAV2 and AAV8 may yield improved variants [51].

An ideal approach would be to identify variants with the ability to reach/target the tissue of interest through manipulation of capsid receptor biology. This variant would then be further modified to account for intracellular trafficking. A method that utilizes directed evolution to find variants with increased affinity for PRs that can subsequently be enhanced by incorporation of the appropriate combination of Y−F and or T−V mutations may ultimately be the most successful strategy, particularly if powerful quantitative assays can be used to rapidly and accurately assess *in vivo* vector properties. We previously described methods for quantifying vector transduction efficiency in a biologically relevant, PR cell line [37]. Here we extend this to a reliable *in vivo* assay for quantifying transduction efficiencies of intravitreally-delivered AAV vectors in mouse PRs. Our quantitative results correlated well to qualitative fundoscopic observations. We demonstrate that quantitative findings could be obtained as early as one week post-injection and that, although fewer total cells appear transduced at this early time point relative to 4 weeks post-injection, the pattern and relative efficiencies of vectors remained the same.

Of all vectors tested, the most robust *in vivo* expression of PRs was noted following intravitreal delivery of AAV2(quadY−F+T−V)−smCBA−GFP. Approximately 22% of PRs expressed detectable levels of transgene following intravitreal injection with this capsid mutant. To what extent transduction of 22% of PRs is capable of preserving retinal structure and/or restoring visual function to an animal model of IRD is yet to be determined. Likewise, whether further improvements in transduction efficiency of the AAV2(quadY−F+T−V) can be achievable by additional mutagenesis requires further investigation. Evidence suggests that directed mutagenesis of additional threnonine, lysine and serine residues, all of which are more abundant on the AAV2 capsid surface than tyrosine, and similarly reduce phosphorylation/proteosomal degradation of capsid, may further augment AAV-mediated transgene expression [29]. It is expected that this approach has a finite maximum. However, it is important to note that the transduction efficiency of capsid mutant vectors varies with the target tissue as well as the profile and activity levels of kinases involved in AAV capsid phosphorylation [52]. Additionally, it has yet to be determined whether initially non surface-exposed residues that become available for phosphorylation in later steps of cellular processing (during conformational changes of the capsid) may also be mutated to improve transduction efficiency.

When considering intravitreal delivery of AAV vector intended to transduce distal PRs, emphasis must be placed on avoiding off-target transgene expression. Consistent with previous reports [18], [31], we found that the hGRK1 promoter drove strong transgene expression in PRs. Unexpectedly, off-target expression was also noted in retinal ganglion cells. Previous studies evaluating GRK1 promoter activity in retina have utilized AAV serotypes with poor tropism for retinal ganglion cells, namely AAV5 and AAV8 [18], [23], [31], [53], [54]. Therefore it is unlikely, even in the event such vectors were delivered to the vitreous, that transduction of retinal ganglion cells would have occurred. When we used a parent serotype with strong affinity for retinal ganglion cells (AAV2) and delivered high titer vector to the vitreous, GRK1 promoter activity in retinal ganglion cells was apparent. Because GRK1 has been shown to promote strong gene expression in both rods and cones of primate retina with no expression in middle retina or retinal pigment epithelium [18] we sought to address specifically the observed expression in retinal ganglion cells. We attempted to reduce this off-target expression by incorporating four tandem sequences complimentary to an inner/middle retina-specific miRNA into our AAV vectors. A microRNA expression atlas of the mouse eye [55] indicates that miR-181c is highly expressed in retinal ganglion cells and middle retina and absent in photoreceptors in P60 mouse (http://mirneye.tigem.it/view_state.php?state=P60&mirna=mmu-miR-181c). Incorporation of miR−181c repeat sequence resulted in ablation of expression in retinal ganglion cells; however it also appreciably reduced expression of transgene in PRs. Attempts are underway to characterize vectors containing fewer miRNA target sequences and/or in a different spatial arrangement with the goal to prevent off-target transduction while preserving PR expression.

An important limitation of any AAV transduction study performed in lower order mammals is its translatability to clinic. The ILM is relatively thin and homogenous in rodents. In primate, the ILM is significantly thicker, except for an area in and around the fovea and immediately above large blood vessels [56]. These enhanced vectors will need to be thoroughly tested to determine if the gains in transduction from the vitreous as seen in mouse translate to similar improvements in the primate retina, particularly the relatively exposed cone-rich fovea.

The work described in this manuscript supports continued development of AAV-based vectors for the treatment of various forms of PR-mediated inherited retinal disease with a surgically less invasive intravitreal injection technique.

## Supporting Information

Figure S1 **Transduction efficiency of scAAV2(quadY-F) and scAAV2(quadY−F+T−V) in PRs of Rho-GFP mice 1 week post intravitreal injection.**
(TIF)

Figure S2 **Representative image of a retinal tissue section from a C57BL/6 mouse injected with AAV2(quadY−F+T−V) (5.0×10^9^ vg delivered), stained for GFP and counterstained with DAPI.** Merged images are presented at 10X to visualize the full retina.
(TIF)

Figure S3 **Fundus image paired with immunohistochemistry of a frozen retinal tissue section from a C57BL/6 mouse taken 4 weeks post-injection with AAV8(doubleY−F+T−V)−CBA−GFP (1.0×10^10^ vg delivered).** A representative 5X magnification is shown for appreciation of entire retina (Panel B). A 20X image around the optic nerve head is shown for visualizing peripapillary expression (Panel C). White arrows demarcate the optic nerve head.
(TIF)

Figure S4 ***In vivo*** **, qualitative analysis of AAV2-based vectors containing the ubiquitous, CBA promoter.** Fundus images paired with immunohistochemistry of frozen retinal cross sections from C57BL/6 mice taken 4 weeks post injection with AAV2(tripleY–F), AAV2(triple Y−F+T−V), AAV2(quadY–F), and AAV2(quad Y−F+T−V) vectors containing ubiquitous promoter CBA driving GFP (1.5×10^10^ vg delivered.) Identical gain and exposures were used for fundoscopy. Retinal sections were imaged at 5X for visualization of the entire retina from periphery to periphery (Panels B,E,H), at 20X for detailed analysis of each retinal cell type (Panels C,F,I) and at 40X for better resolution of outer the retina (insets of Panels C,F,I). All sections were imaged with identical gain and exposure settings. GFP expression is shown in green. Nuclei were counterstained with DAPI (blue).
(TIF)

Figure S5 **Representative image of GFP positive photoreceptors from a mouse injected intravitreally with AAV2(quadY−F+T−V)−CBA−GFP.** Photoreceptors were distinguished from Muller glia processes by counting GFP-positive cell bodies and outer segments (examples demarcated with white arrows).
(TIF)

Figure S6 **Representative image of GFP positive photoreceptors from a mouse injected intravitreally with AAV2(quadY−F+T−V)−hGRK1−GFP.**
(TIF)

Figure S7 **Semi-quantitative comparison of the number of transduced photoreceptors in eyes intravitreally injected with either AAV2(quadY−F+T−V)−hGRK1−GFP or AAV2(quadY−F+T−V)−CBA−GFP.** Photoreceptor transduction was measured as a function of GFP expression in these cells within 4 representative areas of retinas injected with each vector. All areas analyzed were of equal size based on magnification (40X).
(TIF)

## References

[1] Cideciyan AV, Hauswirth WW, Aleman TS, Kaushal S, Schwartz SB, et al (2009) Human RPE65 gene therapy for Leber congenital amaurosis: persistence of early visual improvements and safety at 1 year. Hum Gene Ther 20: 999–1004. 19583479 10.1089/hum.2009.086 PMC2829287

[2] Maguire AM, Simonelli F, Pierce EA, Pugh EN Jr, Mingozzi F, et al (2008) Safety and efficacy of gene transfer for Leber's congenital amaurosis. N Engl J Med 358: 2240–2248. 18441370 10.1056/NEJMoa0802315 PMC2829748

[3] Bainbridge JW, Smith AJ, Barker SS, Robbie S, Henderson R, et al (2008) Effect of gene therapy on visual function in Leber's congenital amaurosis. N Engl J Med 358: 2231–2239. 18441371 10.1056/NEJMoa0802268

[4] Wright AF, Chakarova CF, Abd El-Aziz MM, Bhattacharya SS (2010) Photoreceptor degeneration: genetic and mechanistic dissection of a complex trait. Nat Rev Genet 11: 273–284. 20212494 10.1038/nrg2717

[5] Jacobson SG, Cideciyan AV, Ratnakaram R, Heon E, Schwartz SB, et al (2012) Gene therapy for leber congenital amaurosis caused by RPE65 mutations: safety and efficacy in 15 children and adults followed up to 3 years. Arch Ophthalmol 130: 9–24. 21911650 10.1001/archophthalmol.2011.298 PMC3600816

[6] Jacobson SG, Acland GM, Aguirre GD, Aleman TS, Schwartz SB, et al (2006) Safety of recombinant adeno-associated virus type 2-RPE65 vector delivered by ocular subretinal injection. Mol Ther 13: 1074–1084. 16644289 10.1016/j.ymthe.2006.03.005

[7] Daya S, Berns KI (2008) Gene therapy using adeno-associated virus vectors. Clin Microbiol Rev 21: 583–593. 18854481 10.1128/CMR.00008-08 PMC2570152

[8] Ali RR, Reichel MB, Thrasher AJ, Levinsky RJ, Kinnon C, et al (1996) Gene transfer into the mouse retina mediated by an adeno-associated viral vector. Hum Mol Genet 5: 591–594. 8733124 10.1093/hmg/5.5.591

[9] Auricchio A, Kobinger G, Anand V, Hildinger M, O'Connor E, et al (2001) Exchange of surface proteins impacts on viral vector cellular specificity and transduction characteristics: the retina as a model. Hum Mol Genet 10: 3075–3081. 11751689 10.1093/hmg/10.26.3075

[10] Weber M, Rabinowitz J, Provost N, Conrath H, Folliot S, et al (2003) Recombinant adeno-associated virus serotype 4 mediates unique and exclusive long-term transduction of retinal pigmented epithelium in rat, dog, and nonhuman primate after subretinal delivery. Mol Ther 7: 774–781. 12788651 10.1016/s1525-0016(03)00098-4

[11] Yang GS, Schmidt M, Yan Z, Lindbloom JD, Harding TC, et al (2002) Virus-mediated transduction of murine retina with adeno-associated virus: effects of viral capsid and genome size. J Virol 76: 7651–7660. 12097579 10.1128/JVI.76.15.7651-7660.2002 PMC136354

[12] Acland GM, Aguirre GD, Ray J, Zhang Q, Aleman TS, et al (2001) Gene therapy restores vision in a canine model of childhood blindness. Nat Genet 28: 92–95. 11326284 10.1038/ng0501-92

[13] Vandenberghe LH, Bell P, Maguire AM, Cearley CN, Xiao R, et al (2011) Dosage thresholds for AAV2 and AAV8 photoreceptor gene therapy in monkey. Sci Transl Med 3: 88ra54. 10.1126/scitranslmed.3002103 PMC5027886 21697530

[14] Bennett J, Maguire AM, Cideciyan AV, Schnell M, Glover E, et al (1999) Stable transgene expression in rod photoreceptors after recombinant adeno-associated virus-mediated gene transfer to monkey retina. Proc Natl Acad Sci U S A 96: 9920–9925. 10449795 10.1073/pnas.96.17.9920 PMC22311

[15] Allocca M, Mussolino C, Garcia-Hoyos M, Sanges D, Iodice C, et al (2007) Novel adeno-associated virus serotypes efficiently transduce murine photoreceptors. J Virol 81: 11372–11380. 17699581 10.1128/JVI.01327-07 PMC2045569

[16] Petersen-Jones SM, Bartoe JT, Fischer AJ, Scott M, Boye SL, et al (2009) AAV retinal transduction in a large animal model species: comparison of a self-complementary AAV2/5 with a single-stranded AAV2/5 vector. Mol Vis 15: 1835–1842. 19756181 PMC2743804

[17] Lotery AJ, Yang GS, Mullins RF, Russell SR, Schmidt M, et al (2003) Adeno-associated virus type 5: transduction efficiency and cell-type specificity in the primate retina. Hum Gene Ther 14: 1663–1671. 14633408 10.1089/104303403322542301

[18] Boye SE, Alexander JJ, Boye SL, Witherspoon CD, Sandefer KJ, et al (2012) The Human Rhodopsin Kinase Promoter in an AAV5 Vector Confers Rod- and Cone-Specific Expression in the Primate Retina. Hum Gene Ther 23: 1101–1115. 22845794 10.1089/hum.2012.125 PMC3472519

[19] Stieger K, Colle MA, Dubreil L, Mendes-Madeira A, Weber M, et al (2008) Subretinal delivery of recombinant AAV serotype 8 vector in dogs results in gene transfer to neurons in the brain. Mol Ther 16: 916–923. 18388922 10.1038/mt.2008.41

[20] Mussolino C, della Corte M, Rossi S, Viola F, Di Vicino U, et al (2011) AAV-mediated photoreceptor transduction of the pig cone-enriched retina. Gene Ther 18: 637–645. 21412286 10.1038/gt.2011.3 PMC3131697

[21] Vandenberghe LH BP, Maguire A, Xiao R, McMenamin D, et al.. (2011) Cone and rod transduction with alternative AAV serotypes in the macula of non-human primates. ARVO abstract.

[22] Rabinowitz JE, Rolling F, Li C, Conrath H, Xiao W, et al (2002) Cross-packaging of a single adeno-associated virus (AAV) type 2 vector genome into multiple AAV serotypes enables transduction with broad specificity. J Virol 76: 791–801. 11752169 10.1128/JVI.76.2.791-801.2002 PMC136844

[23] Boye SL, Conlon T, Erger K, Ryals R, Neeley A, et al (2011) Long-term preservation of cone photoreceptors and restoration of cone function by gene therapy in the guanylate cyclase-1 knockout (GC1KO) mouse. Invest Ophthalmol Vis Sci 52: 7098–7108. 21778276 10.1167/iovs.11-7867 PMC3207713

[24] Pang JJ, Dai X, Boye SE, Barone I, Boye SL, et al (2011) Long-term retinal function and structure rescue using capsid mutant AAV8 vector in the rd10 mouse, a model of recessive retinitis pigmentosa. Mol Ther 19: 234–242. 21139570 10.1038/mt.2010.273 PMC3034861

[25] Petrs-Silva H, Dinculescu A, Li Q, Min SH, Chiodo V, et al (2009) High-efficiency transduction of the mouse retina by tyrosine-mutant AAV serotype vectors. Mol Ther 17: 463–471. 19066593 10.1038/mt.2008.269 PMC2835095

[26] Petrs-Silva H, Dinculescu A, Li Q, Deng WT, Pang JJ, et al (2011) Novel properties of tyrosine-mutant AAV2 vectors in the mouse retina. Mol Ther 19: 293–301. 21045809 10.1038/mt.2010.234 PMC3034844

[27] Zhong L, Li B, Mah CS, Govindasamy L, Agbandje-McKenna M, et al (2008) Next generation of adeno-associated virus 2 vectors: point mutations in tyrosines lead to high-efficiency transduction at lower doses. Proc Natl Acad Sci U S A 105: 7827–7832. 18511559 10.1073/pnas.0802866105 PMC2402387

[28] Aslanidi GV RA, Ortiz L, Song L, Ling C, et al. (In press.) Optimization of the capsid of recombinant 1 adeno-associated virus 2 (AAV2) vectors:The final threshold?. PLoS One.

[29] Gabriel N, Hareendran S, Sen D, Gadkari RA, Sudha G, et al (2013) Bio-engineering of AAV-2 capsid at specific serine, threonine or lysine residues improves its transduction efficiency in vitro and in vivo. Hum Gene Ther Methods 10.1089/hgtb.2012.194 PMC3732126 23379478

[30] Wensel TG, Gross AK, Chan F, Sykoudis K, Wilson JH (2005) Rhodopsin-EGFP knock-ins for imaging quantal gene alterations. Vision Res 45: 3445–3453. 16139321 10.1016/j.visres.2005.07.016

[31] Khani SC, Pawlyk BS, Bulgakov OV, Kasperek E, Young JE, et al (2007) AAV-mediated expression targeting of rod and cone photoreceptors with a human rhodopsin kinase promoter. Invest Ophthalmol Vis Sci 48: 3954–3961. 17724172 10.1167/iovs.07-0257

[32] Karali M, Manfredi A, Puppo A, Marrocco E, Gargiulo A, et al (2011) MicroRNA-restricted transgene expression in the retina. PLoS One 6: e22166. 21818300 10.1371/journal.pone.0022166 PMC3144214

[33] Haire SE, Pang J, Boye SL, Sokal I, Craft CM, et al (2006) Light-driven cone arrestin translocation in cones of postnatal guanylate cyclase-1 knockout mouse retina treated with AAV-GC1. Invest Ophthalmol Vis Sci 47: 3745–3753. 16936082 10.1167/iovs.06-0086 PMC1761699

[34] Burger C, Gorbatyuk OS, Velardo MJ, Peden CS, Williams P, et al (2004) Recombinant AAV viral vectors pseudotyped with viral capsids from serotypes 1, 2, and 5 display differential efficiency and cell tropism after delivery to different regions of the central nervous system. Mol Ther 10: 302–317. 15294177 10.1016/j.ymthe.2004.05.024

[35] Zolotukhin S, Potter M, Zolotukhin I, Sakai Y, Loiler S, et al (2002) Production and purification of serotype 1, 2, and 5 recombinant adeno-associated viral vectors. Methods 28: 158–167. 12413414 10.1016/s1046-2023(02)00220-7

[36] Tan E, Ding XQ, Saadi A, Agarwal N, Naash MI, et al (2004) Expression of cone-photoreceptor-specific antigens in a cell line derived from retinal tumors in transgenic mice. Invest Ophthalmol Vis Sci 45: 764–768. 14985288 10.1167/iovs.03-1114 PMC2937568

[37] Ryals RC, Boye SL, Dinculescu A, Hauswirth WW, Boye SE (2011) Quantifying transduction efficiencies of unmodified and tyrosine capsid mutant AAV vectors in vitro using two ocular cell lines. Mol Vis 17: 1090–1102. 21552473 PMC3087449

[38] Al-Ubaidi MR, Matsumoto H, Kurono S, Singh A (2008) Proteomics profiling of the cone photoreceptor cell line, 661W. Adv Exp Med Biol 613: 301–311. 18188958 10.1007/978-0-387-74904-4_35

[39] Klimczak RR, Koerber JT, Dalkara D, Flannery JG, Schaffer DV (2009) A novel adeno-associated viral variant for efficient and selective intravitreal transduction of rat Muller cells. PLoS One 4: e7467. 19826483 10.1371/journal.pone.0007467 PMC2758586

[40] Pulicherla N, Asokan A (2011) Peptide affinity reagents for AAV capsid recognition and purification. Gene Ther 18: 1020–1024. 21490687 10.1038/gt.2011.46 PMC3192935

[41] Li C, Diprimio N, Bowles DE, Hirsch ML, Monahan PE, et al (2012) Single amino acid modification of adeno-associated virus capsid changes transduction and humoral immune profiles. J Virol 86: 7752–7759. 22593151 10.1128/JVI.00675-12 PMC3421647

[42] Pang JJ, Deng WT, Dai X, Lei B, Everhart D, et al (2012) AAV-mediated cone rescue in a naturally occurring mouse model of CNGA3-achromatopsia. PLoS One 7: e35250. 22509403 10.1371/journal.pone.0035250 PMC3324465

[43] Bartel MA, Weinstein JR, Schaffer DV (2012) Directed evolution of novel adeno-associated viruses for therapeutic gene delivery. Gene Ther 19: 694–700. 22402323 10.1038/gt.2012.20

[44] Dalkara D, Kolstad KD, Caporale N, Visel M, Klimczak RR, et al (2009) Inner limiting membrane barriers to AAV-mediated retinal transduction from the vitreous. Mol Ther 17: 2096–2102. 19672248 10.1038/mt.2009.181 PMC2814392

[45] Yin L, Greenberg K, Hunter JJ, Dalkara D, Kolstad KD, et al (2011) Intravitreal injection of AAV2 transduces macaque inner retina. Invest Ophthalmol Vis Sci 52: 2775–2783. 21310920 10.1167/iovs.10-6250 PMC3088562

[46] Summerford C, Samulski RJ (1998) Membrane-associated heparan sulfate proteoglycan is a receptor for adeno-associated virus type 2 virions. J Virol 72: 1438–1445. 9445046 10.1128/jvi.72.2.1438-1445.1998 PMC124624

[47] Chai L, Morris JE (1994) Distribution of heparan sulfate proteoglycans in embryonic chicken neural retina and isolated inner limiting membrane. Curr Eye Res 13: 669–677. 7805398 10.3109/02713689408999903

[48] Akache B, Grimm D, Pandey K, Yant SR, Xu H, et al (2006) The 37/67-kilodalton laminin receptor is a receptor for adeno-associated virus serotypes 8, 2, 3, and 9. J Virol 80: 9831–9836. 16973587 10.1128/JVI.00878-06 PMC1617255

[49] Kaludov N, Brown KE, Walters RW, Zabner J, Chiorini JA (2001) Adeno-associated virus serotype 4 (AAV4) and AAV5 both require sialic acid binding for hemagglutination and efficient transduction but differ in sialic acid linkage specificity. J Virol 75: 6884–6893. 11435568 10.1128/JVI.75.15.6884-6893.2001 PMC114416

[50] Cho EY, Choi HL, Chan FL (2002) Expression pattern of glycoconjugates in rat retina as analysed by lectin histochemistry. Histochem J 34: 589–600. 14626350 10.1023/a:1026032005521

[51] Raupp C, Naumer M, Muller OJ, Gurda BL, Agbandje-McKenna M, et al (2012) The threefold protrusions of adeno-associated virus type 8 are involved in cell surface targeting as well as postattachment processing. J Virol 86: 9396–9408. 22718833 10.1128/JVI.00209-12 PMC3416165

[52] Aslanidi GV, Rivers AE, Ortiz L, Govindasamy L, Ling C, et al (2012) High-efficiency transduction of human monocyte-derived dendritic cells by capsid-modified recombinant AAV2 vectors. Vaccine 30: 3908–3917. 22497875 10.1016/j.vaccine.2012.03.079 PMC3356484

[53] Boye SE, Boye SL, Pang J, Ryals R, Everhart D, et al (2010) Functional and behavioral restoration of vision by gene therapy in the guanylate cyclase-1 (GC1) knockout mouse. PLoS One 5: e11306. 20593011 10.1371/journal.pone.0011306 PMC2892468

[54] Beltran WA, Boye SL, Boye SE, Chiodo VA, Lewin AS, et al (2010) rAAV2/5 gene-targeting to rods:dose-dependent efficiency and complications associated with different promoters. Gene Therapy 17: 1162–1174. 20428215 10.1038/gt.2010.56 PMC2914811

[55] Karali M, Peluso I, Gennarino VA, Bilio M, Verde R, et al (2010) miRNeye: a microRNA expression atlas of the mouse eye. BMC Genomics 11: 715. 21171988 10.1186/1471-2164-11-715 PMC3018480

[56] Matsumoto B, Blanks JC, Ryan SJ (1984) Topographic variations in the rabbit and primate internal limiting membrane. Invest Ophthalmol Vis Sci 25: 71–82. 6199321

