# Correction: Targeting Photoreceptors via Intravitreal Delivery Using Novel, Capsid-Mutated AAV Vectors

**DOI:** 10.1371/annotation/99ee1789-a658-4fb0-8593-40a40e9f344a

**Published:** 2013-09-20

**Authors:** Christine N. Kay, Renee C. Ryals, George V. Aslanidi, Seok Hong Min, Qing Ruan, Jingfen Sun, Frank M. Dyka, Daniel Kasuga, Andrea E. Ayala, Kim Van Vliet, Mavis Agbandje-McKenna, William W. Hauswirth, Sanford L. Boye, Shannon E. Boye

Two additional grants from the National Eye Institute were incorrectly omitted from the Funding Statement. The numbers of the two grants are: R24EY022012 and R01EY017549. 

