# Correction: Targeting Photoreceptors via Intravitreal Delivery Using Novel, Capsid-Mutated AAV Vectors

**DOI:** 10.1371/journal.pone.0110030

**Published:** 2014-09-30

**Authors:** The PLOS ONE Staff

There is an error in Table 1. The values in the first and second rows of the Mutation column are incorrect. Please see the corrected Table 1 here.

**Table 1:** for capsid-mutated vectors with description of amino acid location of mutation.

Vector nomenclature	Mutation
AAV2(tripleY-F)	Y444F+Y500F+Y730F
AAV2(tripleY-F+T-V)	Y444F+Y500F+Y730F +T491V
AAV2(quadY-F)	Y272F+Y444F+Y500F+Y730F
AAV2(quadY-F+T-V)	Y272F+Y444F+Y500F+Y730F+T491V
AAV5(singleY-F)	Y719F
AAV5(doubleY-F)	Y263F+Y719F
AAV8(doubleY-F)	Y447F+Y733F
AAV8(doubleY-F+T-V)	Y447F+Y733F+T494V
