# Supplementary figures and images for: Targeting Photoreceptors via Intravitreal Delivery Using Novel, Capsid-Mutated AAV Vectors
Source: PLoS One. 2013 Apr 26;8(4):e62097. doi: 10.1371/journal.pone.0062097 (PMC3637363; doi:10.1371/journal.pone.0062097)

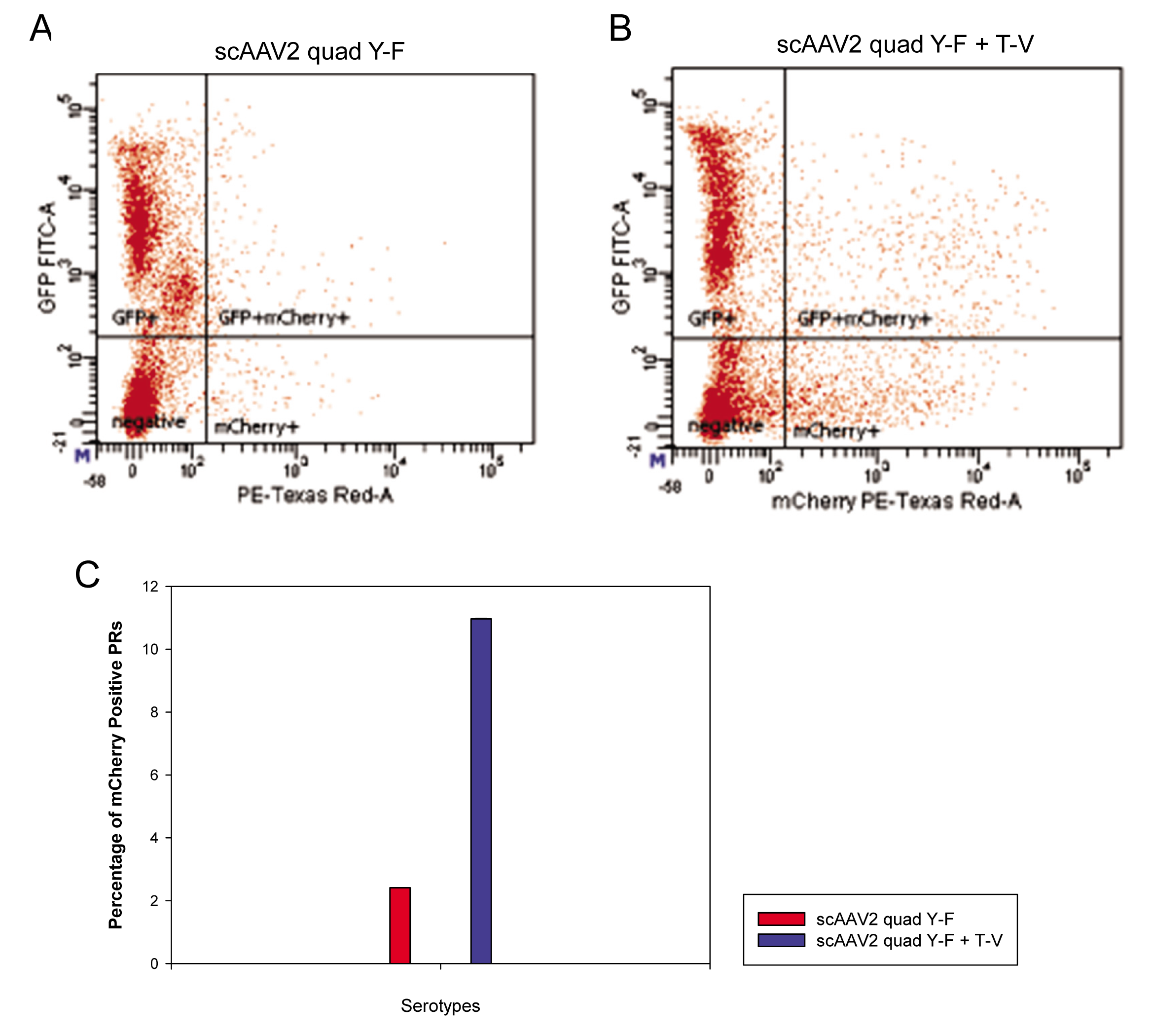

Supplement: Figure S1 — Transduction efficiency of scAAV2(quadY-F) and scAAV2(quadY−F+T−V) in PRs of Rho-GFP mice 1 week post intravitreal injection. (TIF) [file pone.0062097.s001.tif]

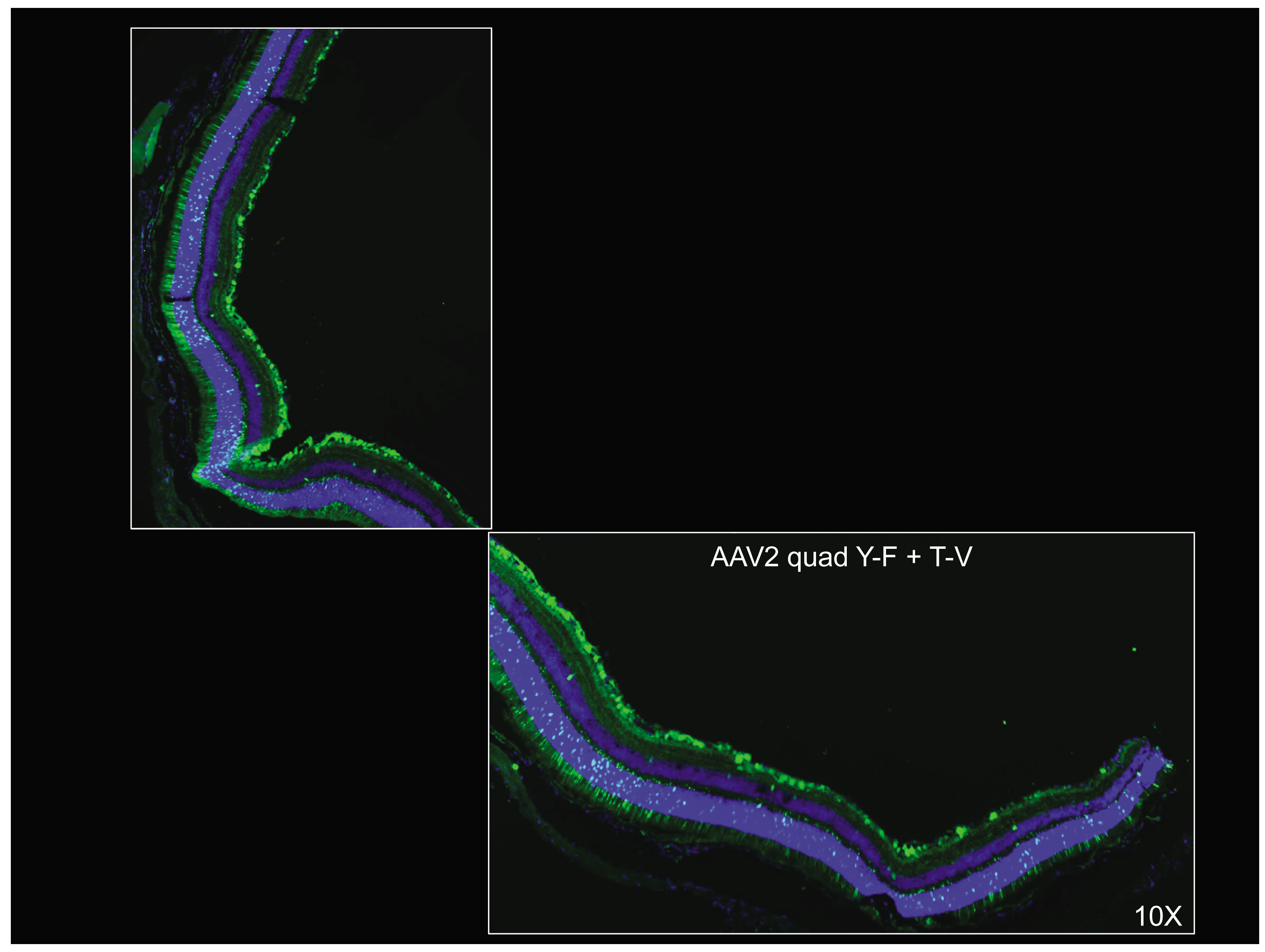

Supplement: Figure S2 — Representative image of a retinal tissue section from a C57BL/6 mouse injected with AAV2(quadY−F+T−V) (5.0×10^9^ vg delivered), stained for GFP and counterstained with DAPI. Merged images are presented at 10X to visualize the full retina. (TIF) [file pone.0062097.s002.tif]

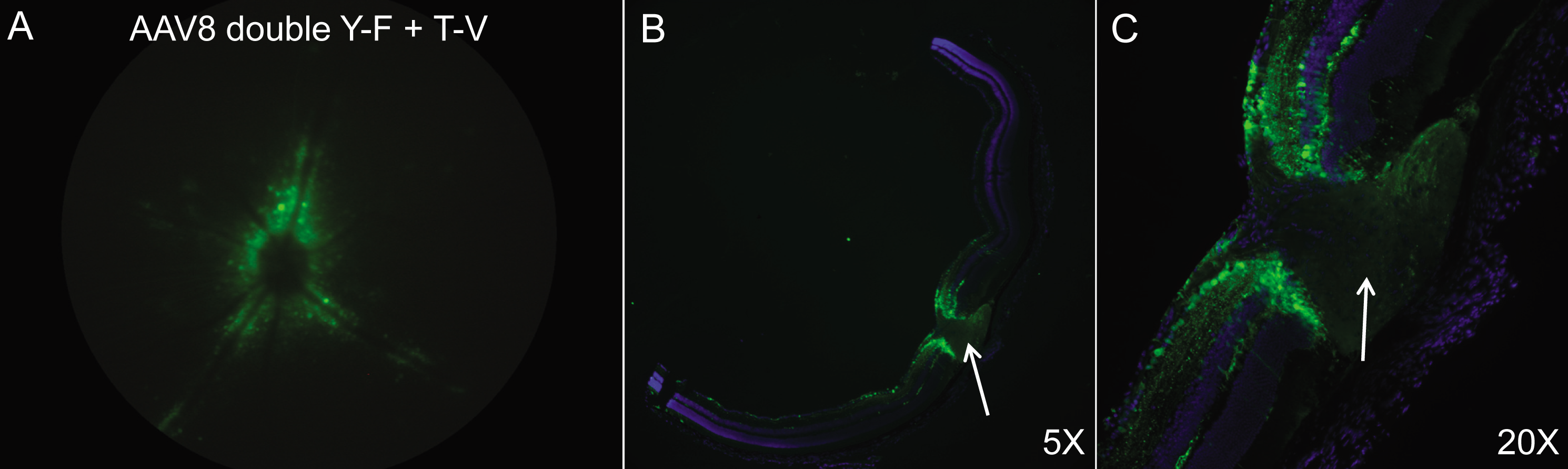

Supplement: Figure S3 — Fundus image paired with immunohistochemistry of a frozen retinal tissue section from a C57BL/6 mouse taken 4 weeks post-injection with AAV8(doubleY−F+T−V)−CBA−GFP (1.0×10^10^ vg delivered). A representative 5X magnification is shown for appreciation of entire retina (Panel B). A 20X image around the optic nerve head is shown for visualizing peripapillary expression (Panel C). White arrows demarcate the optic nerve head. (TIF) [file pone.0062097.s003.tif]

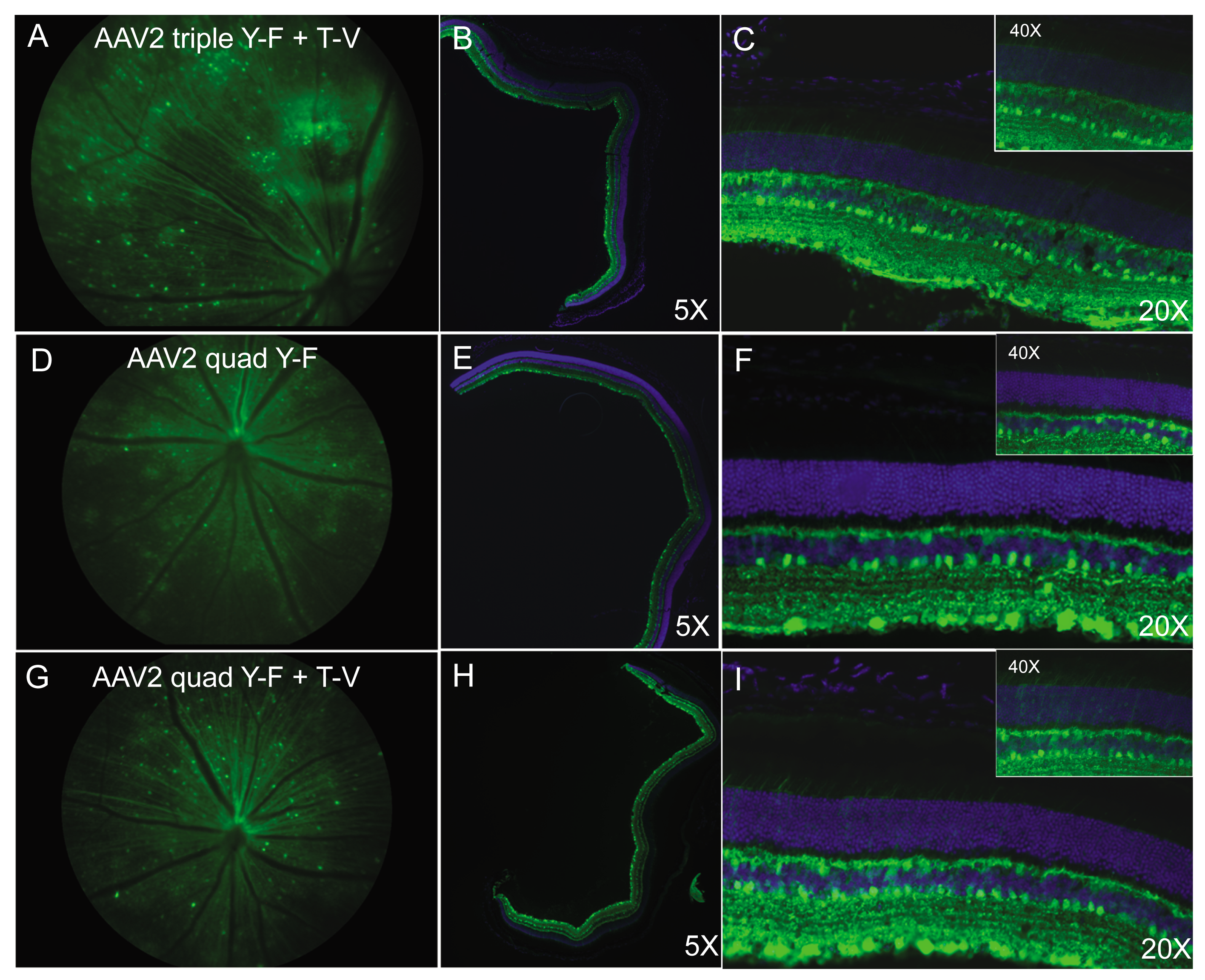

Supplement: Figure S4 — In vivo , qualitative analysis of AAV2-based vectors containing the ubiquitous, CBA promoter. Fundus images paired with immunohistochemistry of frozen retinal cross sections from C57BL/6 mice taken 4 weeks post injection with AAV2(tripleY–F), AAV2(triple Y−F+T−V), AAV2(quadY–F), and AAV2(quad Y−F+T−V) vectors containing ubiquitous promoter CBA driving GFP (1.5×10^10^ vg delivered.) Identical gain and exposures were used for fundoscopy. Retinal sections were imaged at 5X for visualization of the entire retina from periphery to periphery (Panels B,E,H), at 20X for detailed analysis of each retinal cell type (Panels C,F,I) and at 40X for better resolution of outer the retina (insets of Panels C,F,I). All sections were imaged with identical gain and exposure settings. GFP expression is shown in green. Nuclei were counterstained with DAPI (blue). (TIF) [file pone.0062097.s004.tif]

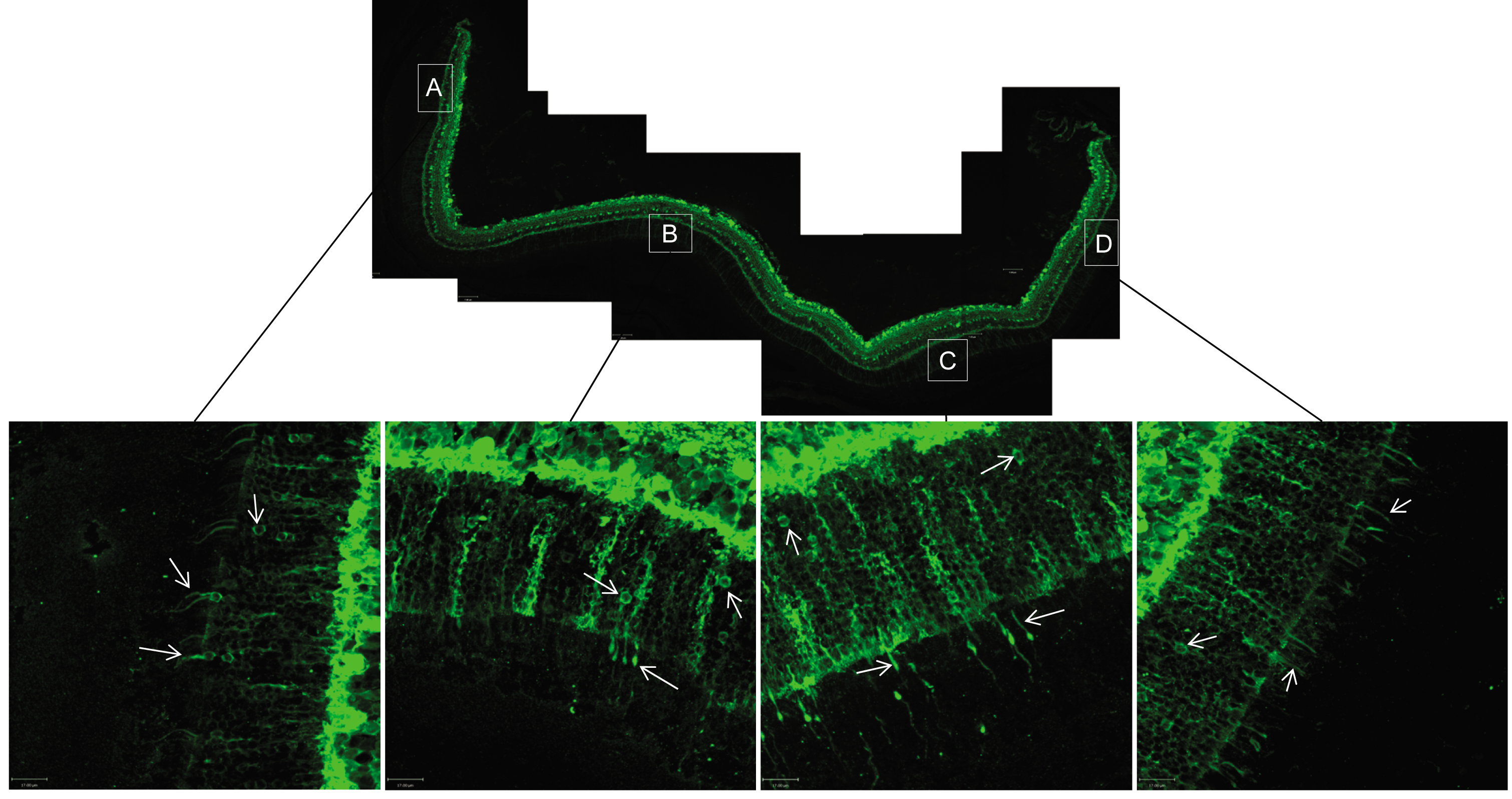

Supplement: Figure S5 — Representative image of GFP positive photoreceptors from a mouse injected intravitreally with AAV2(quadY−F+T−V)−CBA−GFP. Photoreceptors were distinguished from Muller glia processes by counting GFP-positive cell bodies and outer segments (examples demarcated with white arrows). (TIF) [file pone.0062097.s005.tif]

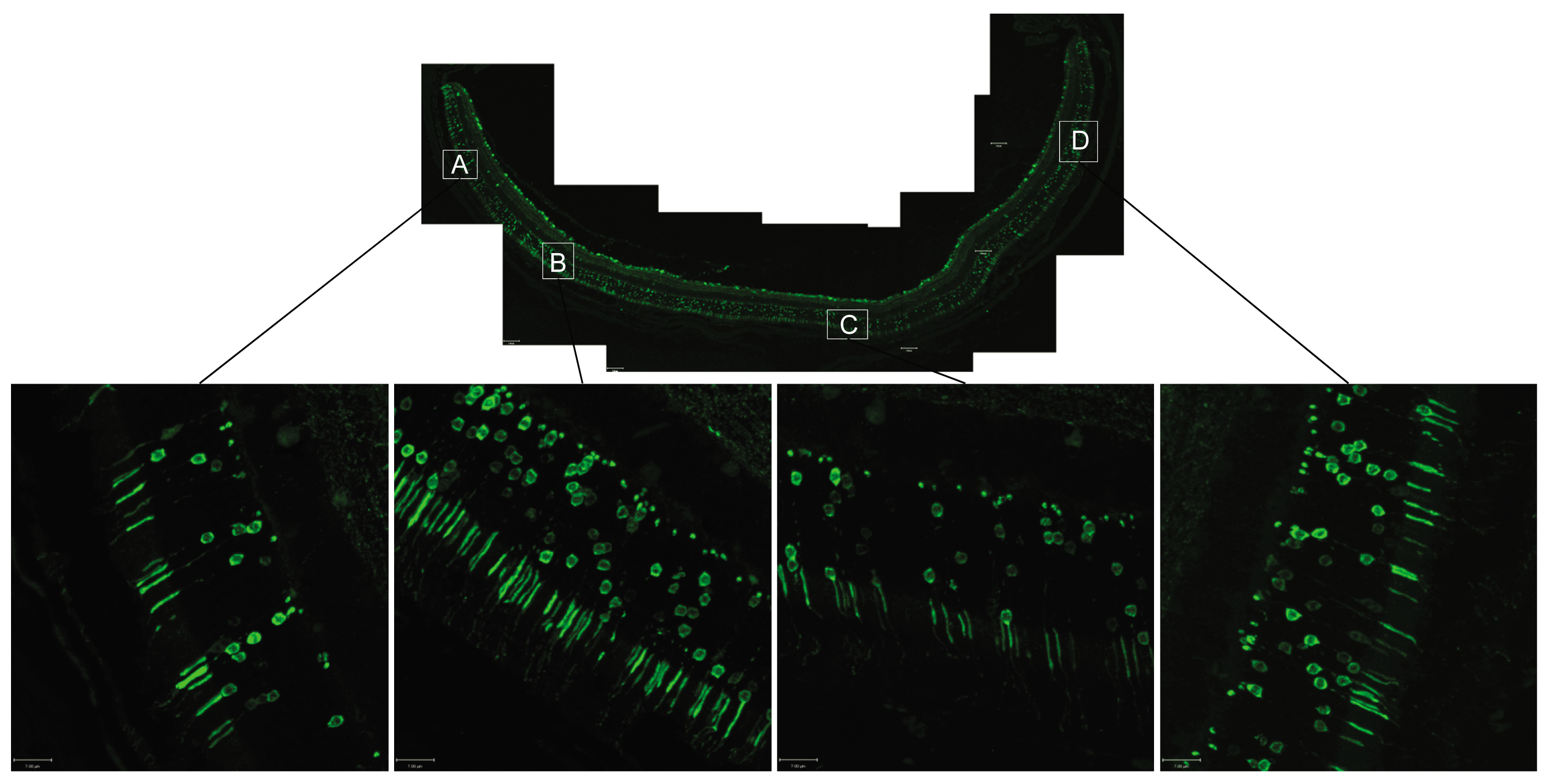

Supplement: Figure S6 — Representative image of GFP positive photoreceptors from a mouse injected intravitreally with AAV2(quadY−F+T−V)−hGRK1−GFP. (TIF) [file pone.0062097.s006.tif]

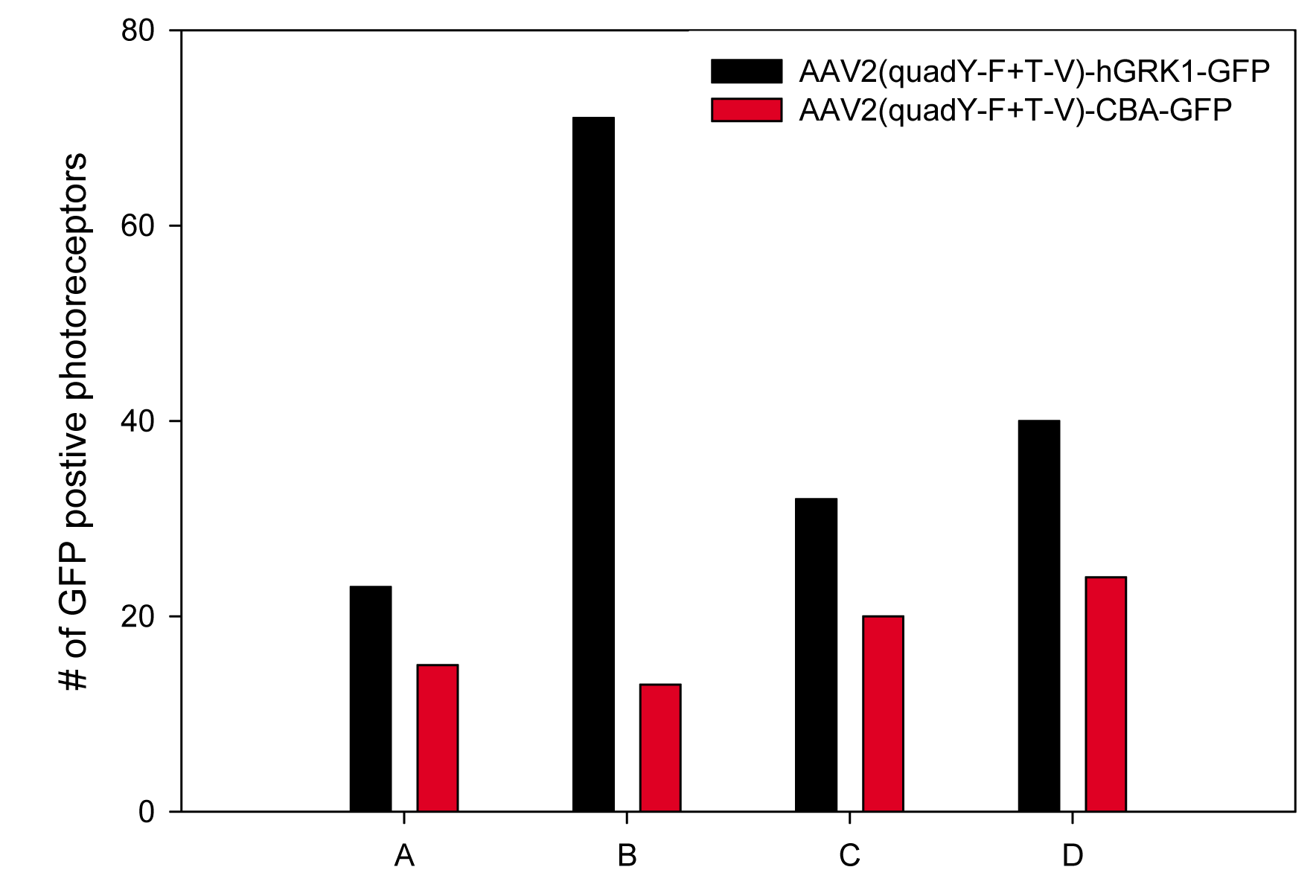

Supplement: Figure S7 — Semi-quantitative comparison of the number of transduced photoreceptors in eyes intravitreally injected with either AAV2(quadY−F+T−V)−hGRK1−GFP or AAV2(quadY−F+T−V)−CBA−GFP. Photoreceptor transduction was measured as a function of GFP expression in these cells within 4 representative areas of retinas injected with each vector. All areas analyzed were of equal size based on magnification (40X). (TIF) [file pone.0062097.s007.tif]
